# Survey of public health education combined with intelligent prescription intervention system on antibiotic use in the county-level hospitals

**DOI:** 10.3389/fpubh.2026.1963236

**Published:** 2026-09-30

**Authors:** Xiaoyan Xie, Lei Yang, Xiaohui Yue, Ailing Ding, Jingjing Zhong

**Affiliations:** 1 Department of Pharmacy, The People’s Hospital of Jianyang City, Jianyang, China; 2 School of Software Engineering, Chengdu University of Information Technology, Chengdu, China

**Keywords:** antibiotics, health literacy intervention, prescription, prescription pre-audit intelligent decision system, rational drug use

## Abstract

**Objective:**

This study evaluated the combined effectiveness of an antibiotic health literacy intervention and a prescription pre-audit intelligent decision system on antibiotic utilization. Thereby providing a reference basis for conducting antibiotic health literacy intervention activities and promoting rational drug use in similar hospitals.

**Methods:**

Evidence-based popular science materials, including handbooks, videos, and lectures, were developed as part of a combined intervention that integrated antibiotic education with a prescription pre-audit intelligent decision system. Variations in irrational antibiotic administration, antibiotic use density, and drug cost ratio were compared between the pre-intervention and post-intervention stages. A retrospective analysis was also conducted to summarize the classification and severity of respiratory ward medication events and the compliance of prescriptions recorded in the system from October to December 2025.

**Results:**

Multiple forms of health literacy intervention materials concerning rational antimicrobial administration were designed in this research, covering printed leaflets, WeChat posts, promotional videos and face-to-face training sessions. Educational interventions combining online and offline modes were associated with reductions in departmental antibiotic use density and the proportion of antibiotic expenditure relative to total medication costs. Integrated with the prescription pre-audit intelligent decision system, prescription-related risks derived from irrational medication orders were stratified into distinct severity grades in light of clinical realities of the ward. From October to December 2025, the counts of prescriptions with defects in treatment indications, dosing schedules, delivery routes and contraindication violations all declined relative to the baseline period of October to December 2024.

**Conclusion:**

This study observed that the multifaceted intervention, including interactive antimicrobial stewardship training for frontline clinicians and nurses, real-time patient-centered health education, and the prescription pre-audit intelligent decision system, has considerable potential to cut antimicrobial consumption. Upgrading health literacy intervention by integrating big data, artificial intelligence and other technologies and extending such comprehensive strategies to the entire hospital represents a promising approach for future implementation.

## 1 Introduction

As the cornerstone for the treatment of bacterial infectious diseases, antimicrobial agents hav (1). Nevertheless, the irrational use and overprescription of antimicrobials have evolved into a critical global public health challenge (2). The World Health Organization (WHO) has categorized bacterial antimicrobial resistance (AMR) as one of the major public health threats to humanity in the 21st century. In 2019, bacterial AMR directly caused 1.27 million deaths worldwide and indirectly contributed to nearly 4.95 million fatalities, with projections indicating that the annual death toll linked to AMR will rise sharply by 2050 (3). Meanwhile, to facilitate global surveillance and stewardship of antimicrobial consumption, the WHO developed the Access, Watch, Reserve (AWaRe) antibiotic classification system, which stratifies systemic antibiotics into three groups according to resistance-selection risk and clinical priority (4). Access antibiotics are preferred first or secondn line agents for common infections with low resistance potential; Watch antibiotics carry higher risks of resistance development and require strict monitoring; Reserve antibiotics are last-reserve options restricted for multi-drug resistant bacterial infections only. WHO has set an international benchmark target that at least 60% of total antibiotic prescriptions should belong to the Access group (5).

A national study conducted in China analyzed more than 170 million outpatient prescriptions from 139 secondary and tertiary public hospitals and found that nearly 11% of these prescriptions contained antibiotics (6). Only 15.3% of such antibiotic prescriptions were rationally prescribed, whereas 51.4% were clearly inappropriate; notably, 4.8% of antibiotic prescriptions showed no correlation with patients’ clinical diagnoses at all. Common forms of antimicrobial overuse include medication without valid indications, inappropriate dosing regimens, and excessive prophylactic administration (7). Such practices not only elevate patients’ risks of adverse drug reactions but also accelerate the emergence and transmission of drug-resistant strains, rendering several conventional antimicrobials ineffective. Accordingly, standardizing antimicrobial utilization has become an urgent public health priority.

From the perspective of medical system hierarchy, county-level hospitals serve as the core provider of county-wide medical services and undertake nearly 70% of medical demands from grassroots populations, representing a critical setting for antimicrobial consumption (8). Compared with large tertiary hospitals, county hospitals are hampered by drawbacks including uneven allocation of medical resources and insufficient professional competency (9). Meanwhile, rural residents and middle-aged and older adult individuals constitute the primary service population of county hospitals. This group generally exhibits inadequate health literacy and harbors widespread misconceptions such as “antimicrobials cure all diseases” and “antimicrobials are mandatory for colds and fevers.” Self-medication as well as arbitrary drug discontinuation and dosage adjustment are also prevalent among them (2). A retrospective cross-sectional study analyzing prescription data of 475 outpatients aged ≥65 years reported that the incidence of potentially inappropriate medication under polypharmacy reached 41.05% (10). In addition, some grassroots physicians rely heavily on empirical treatment, which may lead to excessive prophylactic medication and blind selection of high-grade antimicrobials (11). Such behaviors further exacerbate irrational antimicrobial use, imposing heavier economic burdens on patients and resulting in waste of medical resources. Consequently, county hospitals constitute the weak link in the standardization of antimicrobial utilization.

Among diversified intervention strategies for standardized antimicrobial management, health education intervention stands out as a low-cost, easy-to-implement and replicable vital tool, whose value has been increasingly recognized (12, 13). At present, most interventions targeting standardized antimicrobial use in China focus on clinical in-hospital measures including physician training and prescription review (14, 15). Research regarding supplementary reinforcement via popular science education remains scarce, especially in county-level and grassroots medical settings. The shortage of educational resources and limited communication channels in such areas hinder the correction of public cognitive bias toward antimicrobials.

Against the backdrop of the global initiative to implement the WHO AWaRe antibiotic classification management, all healthcare institutions are encouraged to optimize empirical prescribing for mild infections and raise the proportion of Access group antimicrobial prescriptions to over 60%. According to local guidelines, narrow spectrum antimicrobials in the Access group (e.g., amoxicillin, cefazolin, cephalexin) are prioritized as first-line regimens for empirical management of mild respiratory tract infections. Broad spectrum antimicrobials belonging to the Watch group (e.g., piperacillin-tazobactam, levofloxacin, ceftriaxone, ceftazidime) should only be used when complicated infection is suspected or initial treatment fails (16).

Notably, clinicians’ antibiotic prescribing behavior is not merely determined by their knowledge of prudent antimicrobial use, but also jointly constrained by objective clinical factors including the severity of patient illness (17). Departments such as the respiratory ward in county-level hospitals frequently encounter severe cases, such as acute exacerbations of chronic obstructive pulmonary disease and severe pulmonary infections. For patients with critical conditions, clinicians tend to adopt empiric over-prescribing of antibiotics to avoid the risk of progressive infection, even though they possess adequate knowledge on standardized antimicrobial therapy (18). Pharmacological training targeting physicians alone cannot fully resolve this dilemma. Accordingly, patient-oriented health education interventions carry vital auxiliary value.

Local antimicrobial guidelines serve as the primary reference for clinicians’ routine prescribing. On the premise of adhering to local clinical treatment guidelines, rational selection of antimicrobials should be promoted to gradually meet the WHO-AWaRe management standards. Existing evidence has demonstrated that well-designed and targeted educational interventions can markedly improve public knowledge of antimicrobials, reduce self-medication and overuse, facilitate standardized clinical prescribing practices, and lower the incidence of inappropriate antimicrobial administration (2). Accordingly, this study intends to design an educational intervention based on local antimicrobial guidelines, investigate its impacts on antimicrobial use in county hospitals, and explore health literacy intervention models suitable for primary-care settings. The findings will enrich practical experience of antimicrobial education at primary medical institutions, provide empirical support for county-wide medical facilities to standardize antimicrobial use and curb the progression of AMR, and bear substantial practical and clinical significance for cutting medical expenditure and safeguarding the health of grassroots residents (Figure 1).

**Figure 1:**
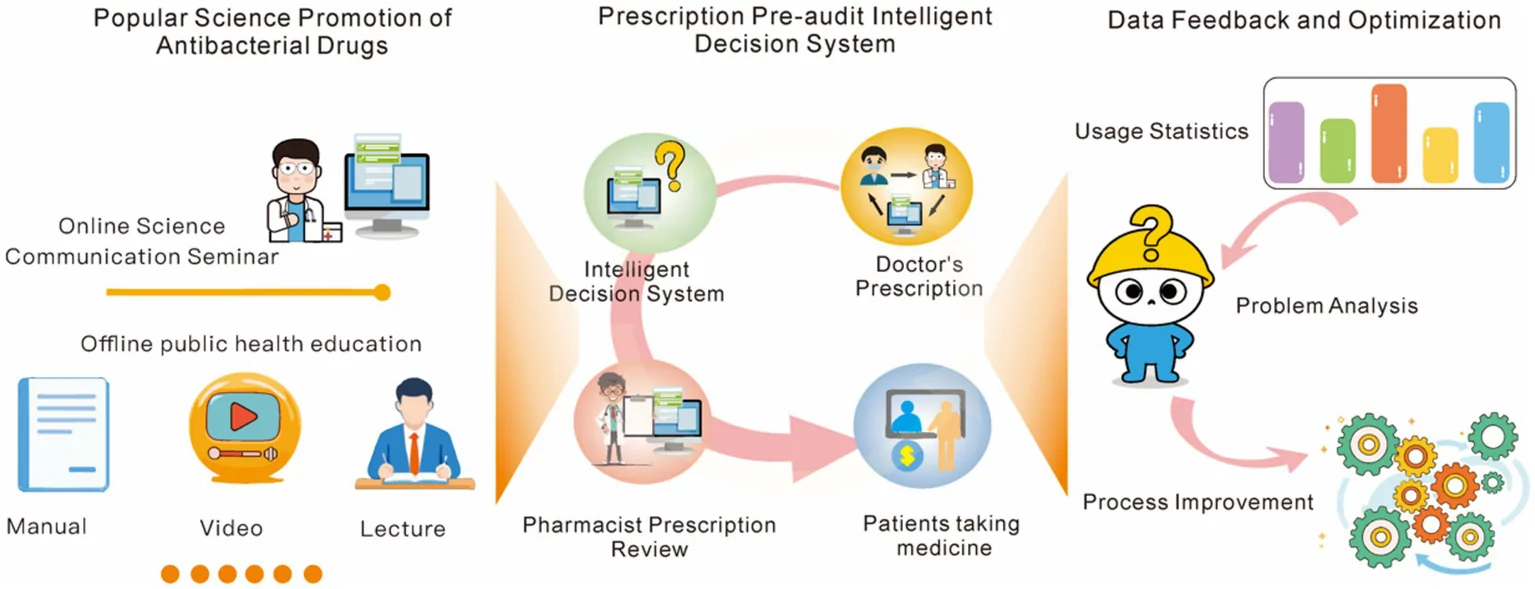
Schematic diagram of health popularization intervention. Multiple online and offline educational approaches were adopted to implement intervention based on the Pre-prescription Intelligent Decision System (PPIDS). Relevant triggering rules were optimized and upgraded to further guarantee intervention efficacy.

## 2 Methodology

### 2.1 Subjects

The Department of Respiratory Medicine of a county-level general hospital was selected as the research site, which consumes large quantities of antimicrobials and manages numerous respiratory infections including AECOPD and CAP. Owing to a substantial COPD patient population, many patients believe that infections require intravenous antibiotics, rendering this setting ideal for physician patient two way health education. Moreover, most respiratory inpatients and outpatients are local middle-aged and older adult people, well aligned with our study’s target population. We used a cross-year same-season before-and-after design with baseline (The fourth quarter of 2024) and intervention (The fourth quarter of 2025) periods, enrolling respiratory inpatients and outpatients in both phases. Inclusion criteria: age ≥18 years, absence of severe hepatorenal insufficiency, mental illness or cognitive impairment. Exclusion criteria: inpatients with immunodeficiency or long-term immunosuppressant treatment. Only aggregated department prescription data were collected at the fourth quarter of 2024, with no interventions. The 3-month combined health education and intelligent-tool intervention was performed alongside data collection in the fourth quarter of 2025. Identical eligibility criteria were applied to the 2024 baseline and 2025 intervention cohorts.

Furthermore, it should be further noted that the inclusion and exclusion criteria were only used for individual case analysis of prescription appropriateness. Antibiotic Use Density (AUD) and antimicrobial cost proportion are aggregated ward-level metrics derived from all hospitalized patients in the respiratory ward, including patients with immunodeficiency and long-term immunosuppression. The before-after comparison thus reflects the true antimicrobial consumption and economic burden within the department. The formula is defined as: AUD = (Σ[antibiotic sales (g) / WHO DDD] × 100)/Patient-Days, DDD: Defined Daily Dose; Patient-Days: the total number of days that patients were hospitalized during the same period.

### 2.2 Evaluation of prescription appropriateness

For enrolled hospitalized patients with multiple modifications of empirical antibiotic regimens, each therapeutic switch was assessed independently. The rationality of initial empirical therapy and all subsequent antibiotic replacements was evaluated separately, based on national antimicrobial guidelines, local hospital policies and WHO AWaRe stewardship recommendations. The assessment items included valid indications for regimen change, appropriateness of antibiotic selection, dosage and administration route, and unwarranted escalation to Watch group broad spectrum agents for mild infections. A single case was classified as irrational prescribing if any round of antibiotic replacement was assessed as inappropriate.

### 2.3 Methods of health literacy intervention

The fourth quarter is the peak period of respiratory infections owing to lower temperatures. Seasonal confounders exert marked impacts on respiratory case load, spectrum of infectious diseases and antimicrobial prescribing patterns. To match seasonal, climatic and epidemic conditions of respiratory illnesses, reduce bias from seasonal variation and objectively assess the real effect of the health education intervention, we performed a cross-year comparison between the fourth quarter of 2024 and the fourth quarter of 2025. Changes in the incidence of irrational clinical antimicrobial use, antimicrobial use density and drug cost ratio among study subjects were compared pre- and post-intervention to evaluate the efficacy of the educational intervention. Indicators including the irrational rate of clinical antimicrobial prescriptions and antimicrobial use density were statistically calculated as baseline data before intervention. During the intervention period, systematic antimicrobial popular science education was delivered to all enrolled patients, while clinicians and pharmacists participated in the implementation and guidance of the intervention program throughout the whole stage. At the follow-up phase for efficacy assessment, all post-intervention indicator data were collected. By comparing the discrepancies between pre-intervention baseline data and post-intervention follow-up data, the impacts of health education intervention on antimicrobial utilization in county-level hospitals were clarified, and the overall intervention outcomes were assessed.

Detailed intervention measures were listed as follows: (1) Offline health literacy intervention lectures: One lecture regarding rational antimicrobial use was delivered monthly in the hospital outpatient hall and inpatient wards. The contents covered basic concepts of antimicrobials, applicable indications, hazards of overuse, standard administration regimens, knowledge of antimicrobial resistance, and common cognitive misconceptions. Lectures were illustrated with typical clinical cases, followed by interactive Q&A sessions and distribution of educational brochures. (2) Individualized health guidance: During diagnosis and treatment, medical staff provided one-on-one education tailored to the specific conditions and medication demands of patients in the intervention group. Clinicians emphasized the therapeutic effects, dosage regimens, treatment courses, precautions of prescribed antimicrobials, as well as risks of arbitrary dose adjustment or premature discontinuation, so as to correct inappropriate medication perceptions. (3) Multi-media popular science dissemination: Multiple platforms including the hospital official WeChat account, video channel, outpatient electronic screens and ward publicity boards were utilized to regularly release antimicrobial education articles, animated videos and posters. The materials were updated 1 to 2 times per week for convenient access by patients and their family members. (4) Special consultation desks were established at outpatient and inpatient pharmacies, where clinical pharmacists answered inquiries about antimicrobial administration raised by patients and relatives. (5) Synchronized education for family members: For older adult patients and those with low educational attainment, synchronous health education was provided to their caregivers. Family members were guided to supervise standardized medication, thereby constructing a three-party collaborative intervention model consisting of medical staff, patients and their relatives.

### 2.4 Evaluation indicators of intervention efficacy

Cooperating with the hospital Quality Control Department, clinical pharmacists calculated the prevalence of irrational antimicrobial prescriptions via the Pre-prescription intelligent decision system (PPIDS)[14] (12). This information tool was locally implemented and clinically validated in our hospital, and has been routinely applied for pharmaceutical quality-control practices. Embedded with a rule-based library, the system can automatically identify inappropriate prescription patterns including non-indicated medication, inappropriate drug selection, and flawed dosing regimens. Essentially, every prescription generated following patient consultation is subject to review by the PPIDS. Approved prescriptions allow patients to complete payment and drug dispensing, whereas those failing review must receive dual-signature confirmation or revision prior to any subsequent processing. Clinical pharmacists further performed manual review and verification based on the system’s preliminary screening results to obtain the incidence of inappropriate antimicrobial prescriptions. The inappropriate prescription types included mismatched indications, improper dosage regimens, incorrect administration routes, unreasonable treatment courses and irrational combined medication regimens. The irrational prescription rate of antimicrobials was computed accordingly. Meanwhile, core indicators before and after intervention were statistically collected, such as AUD and the proportion of antimicrobial expenditure in the total pharmaceutical cost.

### 2.5 Design of health literacy intervention

The respiratory ward mainly admits older adult patients with chronic diseases, primarily diagnosed with COPD, asthma, infections and other respiratory diseases. These patients often have long disease courses and impaired immunity, leading to frequent antimicrobial consumption. Most participants are older adults with high comorbidity rates; hypertension, diabetes and coronary heart disease are the most prevalent. Antimicrobial treatment for this group needs to consider underlying chronic conditions, which greatly complicates dosing regimens. Hence, patients and family caregivers urgently require standardized rational drug education. We designed targeted educational materials including health literacy intervention leaflets, WeChat popular science articles, and health education videos. Standardized training regarding antimicrobial use was also delivered to clinicians. Regular targeted training enhances clinicians’ rational prescribing awareness and standardizes antimicrobial practice. This intervention can improve clinical decision-making, cut inappropriate prescriptions, mitigate bacterial resistance risks, and ensure medical quality and patient safety.

### 2.6 Statistical analyses

Data processing were performed using GraphPad Prism 9.0 software. The *t*-test was applied for measurement data. Rates were reported for categorical data, with *χ^2^* tests applied for intergroup comparisons. *p* value < 0.05 was considered statistically significant.

### 2.7 Ethics statement

This study protocol was approved by the Ethics Committee of Jianyang People’s Hospital (ethical approval number: JYL2024004Z).

## 3 Results

### 3.1 Reach of the educational campaign

This study designed multiple forms of health literacy interventions to deliver public health education for clinicians and patients. In total, three special lectures for clinical physicians were held, with approximately 60 attendances. In addition, 10 leaflets were designed (Supplementary Figures 1, 2), and around 600 printed leaflets on rational antimicrobial use were distributed. For online dissemination, six WeChat official account articles were prepared (Supplementary Figure 3), achieving a total of approximately 3,000 views. Four animated videos were produced (Supplementary Figure 4), with a cumulative view count of around 500.

### 3.2 Level analysis of triggering questions

Through the combined intervention of public education and the prescription pre-review system, we analyzed the triggered alert levels and the predominant alert types in respiratory medicine prescriptions between the fourth quarter of 2024 and the fourth quarter of 2025 (Figure 2; Tables 1, 2). The analysis revealed that the number of alerts at Level 5 decreased significantly, with their proportion dropping from 44.13‰ to 19.52‰, whereas those at Levels 6–8 increased markedly (Figure 2A; Table 1; Supplementary Table 1), suggesting that after refining the rule settings within the rational drug use system—owing to the optimization of the rule tree and the more granular specification of drug details—the frequency of rule-triggered information detected by physicians further increased, indicating that the rational drug use system plays a key role in promoting appropriate medication use. Moreover, among the alert types generated by the system, the number of triggers related to dosage and administration (from 17.63‰ to 10.93‰), route of administration (from 11.24‰ to 2.33‰), and contraindications (from 12.21‰ to 5.96‰) decreased significantly compared with the fourth quarter of 2024 (Figure 2B; Table 2; Supplementary Table 2). These findings demonstrate that the integrated strategy of public education combined with the prescription pre-review system has achieved notable effectiveness in improving prescription interventions in the outpatient and emergency settings of respiratory medicine. During the study period, continuous refinements to the alert system’s sensitivity and rule-tree framework may introduce measurement bias in comparisons of alert frequency. Thus, this issue should be fully considered when interpreting the results.

**Figure 2:**
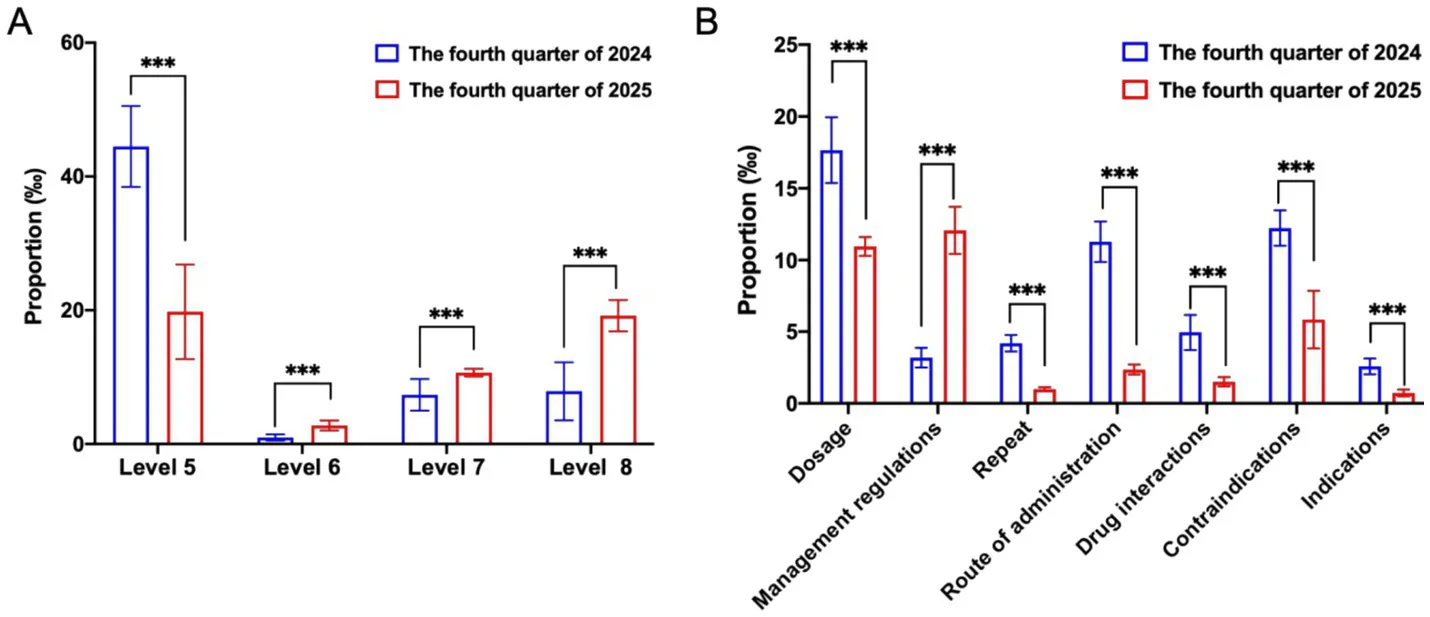
Proportions of prescription alert levels and types in respiratory medicine outpatient clinics before and after the intervention. **(A)** Pre-audit Intelligent Decision System intervention alert level 5–8, Level 5: advisory messages to clinicians for potential medication errors. Level 6: require clinician dual signature. Level 7: mandate revision of detected prescription errors before saving. Level 8: block prescription submission, as definite errors prohibit prescription issuance. **(B)** Pre-audit Intelligent Decision System intervention alert types. Statistical significance was calculated via *χ^2^* test, ***p < 0.001.

**Table 1:** Proportions of PPIDS alert level 5–8 before and after intervention.

Alert Levels	Before intervention (‰, prescriptions/total prescriptions)	After intervention (‰, prescriptions/total prescriptions)	*χ^2^*	*p*
Level 5	44.13(911/20642)	19.52(436/22333)	292.67	<0.001
Level 6	0.96(20/20642)	2.73(61/22333)	22.74	<0.001
Level 7	7.41(153/20642)	10.61(237/22333)	13.41	<0.001
Level 8	7.99(165/20642)	18.98(424/22333)	112.81	<0.001

**Table 2:** Proportions of different PPIDS alert types before and after intervention.

Alert types	Before intervention (‰, prescriptions/total prescriptions)	After intervention (‰, prescriptions/total prescriptions)	*χ^2^*	P
Dosage	17.63 (364/20642)	10.93 (244/22333)	34.61	<0.001
Management regulations	3.2 (66/20642)	11.96 (267/22333)	107.02	<0.001
Repeat	4.21 (87/20642)	0.99 (22/22333)	44.23	<0.001
Route of administration	11.24 (232/20642)	2.33 (52/22333)	129.75	<0.001
Drug interactions	4.89 (101/20642)	1.48 (33/22333)	40.25	<0.001
Contraindications	12.21 (252/20642)	5.96 (133/22333)	47.24	<0.001
Indications	2.62 (54/20642)	0.72 (16/22333)	23.79	<0.001

### 3.3 Analysis of antimicrobial use

In addition, we analyzed medication-related data from respiratory inpatients. Unfortunately, there are substantial discrepancies between China’s national catalog of unrestricted-class antimicrobials and the Access group in the WHO AWaRe classification (19). Therefore, we have not performed stratified analysis for this category, and this limitation should be noted during data interpretation. On the other hand, the drug cost ratio and AUD decreased (*p* = 0.023) after the public education intervention (Figure 3; Table 3; Supplementary Table 3). Notably, the use densities of both Watch group [(21.18, 58.86), *p* = 0.012] and Reserve group [(17.98, 42.91), *p* = 0.009] antimicrobials also decreased (Figures 3C,D). This improvement may be attributable to the fact that, while developing the educational materials, our research team may have exerted a subtle, yet positive, influence on the prescribing behaviors of healthcare professionals. Alternatively, the enhanced disease awareness among patients, resulting from the educational campaign, may have positively contributed to the acceptance and adherence to physicians’ therapeutic regimens.

**Figure 3:**
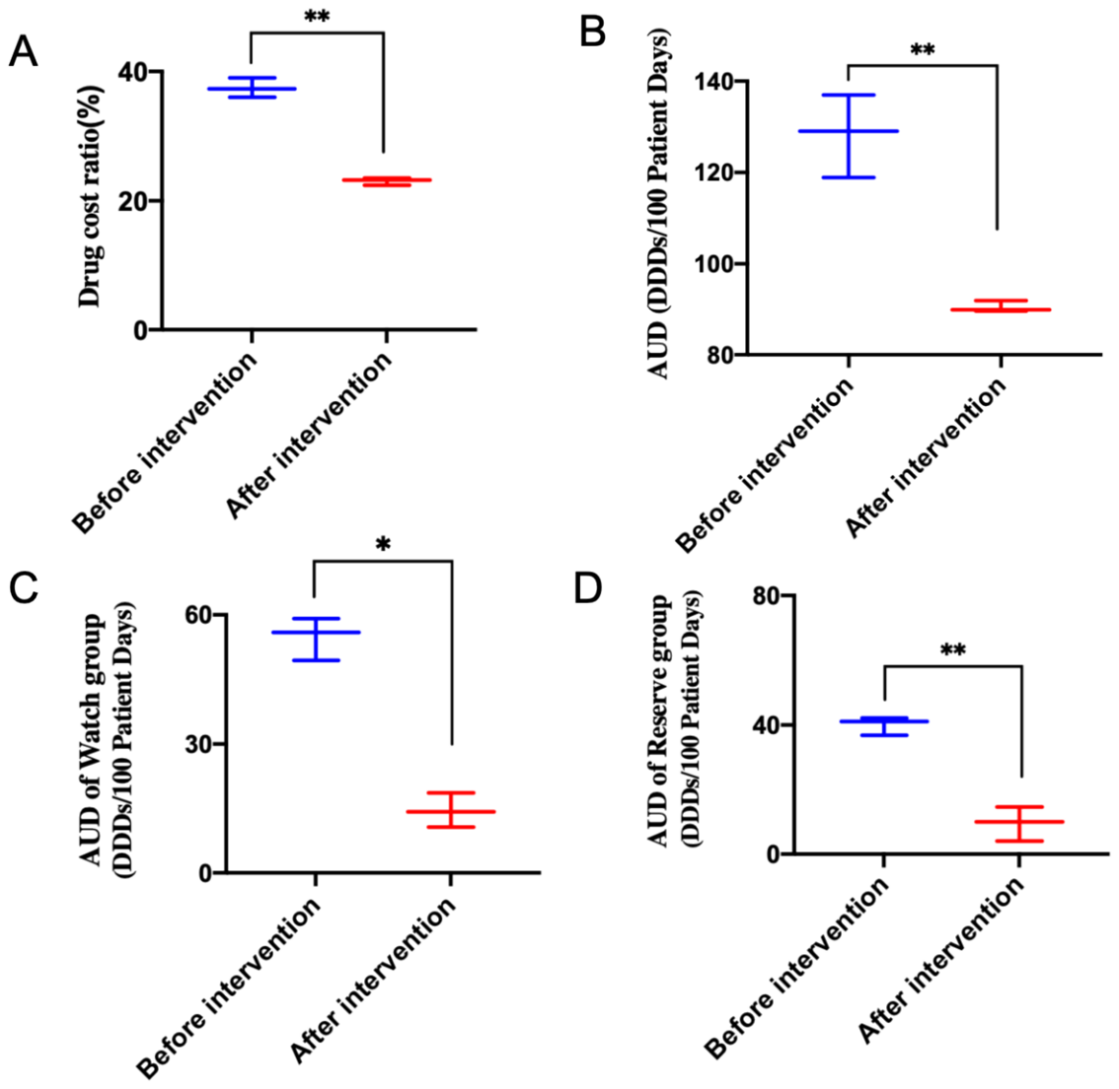
Drug cost ratio and antimicrobial use in respiratory inpatients before and after intervention. **(A)** Drug cost ratio before and after intervention. **(B)** AUD before and after intervention. **(C)** Consumption density of Watch group before and after intervention (e.g., Ceftazidime, Ceftriaxone, Piperacillin-tazobactam). **(D)** Consumption density of Reserve group before and after intervention (e.g., Meropenem, Imipenem-cilastatin, Vancomycin). Statistical significance was calculated via t-test, *p < 0.05, **p < 0.01.

**Table 3:** The statistical analysis of AUD values before and after the intervention.

AUD	Before intervention		After intervention		95% CI	Effect sizes	*p*
	Mean ± SD	IQD	Mean ± SD	IQD			
AUD of Antibiotics(DDDs)	128.30 ± 9.08	9.05	90.43 ± 1.37	0.26	[12.83, 62.92]	3.76	0.023
AUD of Watch(DDDs)	54.84 ± 4.87	4.85	14.82 ± 4.06	4.06	[21.18, 58.86]	5.28	0.012
AUD of Reserve group(DDDs)	40.02 ± 2.70	0.52	9.57 ± 5.43	5.72	[17.98, 42.91]	6.07	0.009

SD, Standard deviations; IQR, Interquartile ranges; 95% CI, 95% confidence intervals.

## 4 Discussion

Our study demonstrates that health education strategies disseminating knowledge about disease recognition, antibiotic selection, and associated risks can be successfully implemented in primary-level hospitals and yield favorable outcomes, which is consistent with findings from pilot studies conducted on single floors in academic medical centers and community hospitals (20, 21). As rational antibiotic prescribing improved, AUD for Watch and Reserved category antimicrobials declined post-intervention, showing a synchronous trend between better prescription appropriateness and reduced AUD of Watch and Reserved agents. Watch and Reserved antimicrobials are mostly broad-spectrum drugs with high selective pressure and are the main contributors to inappropriate antimicrobial use in the respiratory ward of primary hospitals. Rapid emergence of multidrug-resistant organisms is frequently linked to high utilization of Watch and Reserved antimicrobials in daily clinical care. Following our bidirectional doctor-patient health education intervention, clinicians reduced unnecessary empirical prescriptions of these agents and prioritized safe and effective Access category antimicrobials, aligning with WHO AWaRe global targets. Furthermore, this implementation effort provided us with an opportunity to evaluate how this combination of health education interventions is influenced by different clinical practices and cultural contexts, how the roles and responsibilities of various stakeholders should be defined, and how workflows can be standardized to achieve consistent implementation. These insights are of critical importance for the future scaling-up of this approach within county-level medical consortium centers.

During the implementation of the health education strategy, we primarily focused on two target groups: physicians and patients. Prior to delivering the educational interventions, we first provided training to frontline physicians on the importance of appropriate antibiotic use. Subsequently, patient education was conducted. Nevertheless, public health education in this study targeted middle-aged and older adult patients at county hospitals. Differences in patients’ educational background directly influence the performance of educational interventions (22, 23). Patients with low literacy have poor antimicrobial knowledge and limited acceptance of text-heavy professional materials, and benefit more from accessible formats including posters, face-to-face counseling and short videos. Patients with higher education can obtain information through brochures and popular science WeChat articles. Furthermore, given that the specific components of the patient education package needed to be freely and publicly accessible, we utilized a standardized toolkit, including leaflets, posters, videos, and PowerPoint slides, which played a key role during the engagement, education, and execution phases of the intervention.

The PPIDS contributes substantially to improving patient medication safety and rational medication use at our hospital (14). Nevertheless, the system requires fine-tuning and ongoing updates to its rule base to enable effective prescription review and improve prescription assessment accuracy. Notably, no targeted intensive antimicrobial education for the respiratory department and no regular feedback and review of alert data were performed at baseline. The 3-month (one-quarter) intervention included specialist training for clinicians and patient-oriented health education. We strengthened the use of the existing intelligent system, regularly summarized alert data and fed results back to clinicians, so that technical tools and humanistic education could work synergistically. The intelligent system provides immediate alerts for inappropriate prescriptions to restrict irrational prescribing in real time. However, pop-up alerts alone may be ignored by clinicians, resulting in declining compliance. Sustained educational support is necessary to gradually modify clinicians’ prescribing patterns and patients’ medication expectations. Such cognitive and behavioral changes take time. Hence, we adopted a 3-month intervention period to capture measurable changes in outcomes. This duration is not universally mandatory; hospitals may tailor the intervention length based on their baseline inappropriate prescription rates. Prior studies indicate that electronic decision-support tools combined with bidirectional doctor-patient education yield sustained antimicrobial prescribing improvements, as alerts alone fade over time and education maintains initial benefits (11, 24).

Combined with the PPIDS and bidirectional doctor-patient health education, these two approaches synergistically reduced the consumption of Watch and Reserved category antimicrobials under the WHO AWaRe classification. Health education mitigated patients’ inappropriate requests for broad-spectrum antibiotics, eased clinicians’ clinical burden, and promoted the preference for Access agents. The intelligent prescription system delivered real-time pop-up warnings for inappropriate Watch/Reserve prescribing, limiting overuse of broad-spectrum, high-resistance-risk antimicrobials. However, these interventions can only guide and modulate prescribing behavior and cannot bring about total prescription category shift. Watch and Reserved drugs remain essential for patients with definite indications including severe or suspected resistant infections. Neither digital tools nor training should rigidly mandate universal switching to Access agents, clinical judgment must always center on individual patient status.

In this study, the intelligent pre-prescription tool provided immediate technical constraints; however, over-reliance on system pop-up alerts may lead to alert fatigue (24). Public health education played a more critical driving role. By strengthening clinicians’ prescribing knowledge and correcting patients’ inappropriate demands simultaneously, it relieved demand-side pressure for inappropriate antimicrobial use at primary care settings and consolidated the intervention effects of intelligent tools (25). Intelligent tools deliver passive reminders and hardly change the ingrained perceptions of doctors and patients, whereas public health education enables in-depth cognitive transformation, serving as the core foundation for sustained improvements in prescribing practice (26). Therefore, continuous strengthening of public health education is essential to promote rational medication use among patients.

This study possesses several advantages in terms of intervention implementation and trial design. The intervention protocol was grounded in a proactive implementation framework, employing a multidimensional stratified intervention approach coupled with continuous optimization and iterative refinement, thereby ensuring that the intervention measures remained aligned with clinical practice and were dynamically improved. By integrating health education interventions with a prescription intelligent review system, we enabled automated data extraction and real-time intervention. Furthermore, the study adopted a dual-dimensional management strategy targeting both physicians and patients: patient education was complemented by specialized anti-infective training for physicians, which continuously reduced the rate of inappropriate antimicrobial use and the risk of bacterial resistance. Notably, the intervention adherence was high; the use of plain-language educational content, as opposed to rigid regulatory directives, was more readily accepted by middle-aged and older adult patients in primary care settings, which is conducive to achieving sustainable and long-term refinement of antimicrobial stewardship in the future (27).

Several potential limitations should be considered when interpreting the findings of this study. First, our analysis only evaluated the overall outcomes of the health education intervention, and detailed data regarding the specific educational content received by individual patients were not collected. Owing to the lack of individual demographic data, baseline characteristics of patients before and after intervention could not be balanced or compared, and unmeasured confounding caused by shifts in case composition cannot be fully ruled out. Consequently, we were unable to distinguish the intervention effects attributable to different formats, such as printed leaflets, Animiz-based educational animations, and face-to-face health education-nor could we quantify the independent contribution of any single educational modality in correcting inappropriate antimicrobial use. However, it should be noted that this combined intervention approach, which allowed patients to choose their preferred communication channels, was a key factor in the successful implementation of the program. Second, the health education intervention was delivered solely in the respiratory department, and stratified data on patients’ comorbidities were not collected. The existing data do not allow stratified subgroup analyses. Hence, we cannot ascertain the differential benefits of this intervention across different disease populations. As a relative metric, antimicrobial cost proportion is influenced by confounders including shifts in non-antimicrobial consumption and price changes from national volume-based procurement, and this point should be noted when interpreting the results. Notably, the small sample size precludes clear evaluation of normality. Although the difference values are roughly symmetric in distribution shape, central tendency and dispersion and generally meet the robustness assumption of the t-test regarding normality, we acknowledge the limitations of this statistical approach. Furthermore, this study is limited by the lack of a true concurrent parallel control design. Temporal confounding cannot be fully excluded: clinicians’ stewardship awareness and patients’ health literacy may improve spontaneously over time or via routine hospital quality-control activities. While inter-group t-test partially reduced such bias, time-driven influences could not be completely eliminated. Future studies should adopt rigorous concurrent parallel-control designs to validate the real effect of this educational intervention. Furthermore, our study design cannot separate the independent effects of patient/physician education from automated software hard-stops or alerts. Meanwhile, temporal confounders (including changes in hospital administrative policies and seasonal shifts between viral and bacterial infections) and the Hawthorne effect also cannot be excluded. No validated prospective screening scale was used. Retrospective electronic medical records review excluded documented cognitive/psychiatric cases, missing undiagnosed subclinical ones and causing potential bias.

Although these limitations hinder the refinement and targeted optimization of the intervention program, they reflect the common reality of county-level hospitals. This contextual similarity actually enhances the generalizability of our findings to similar primary care institutions and does not invalidate the overall effectiveness of the health education intervention in promoting appropriate antimicrobial use.

## 5 Conclusion

This study implemented a 3-month intervention combining clinician-patient health education and PPIDS. The results suggest that it improved the appropriateness of antimicrobial prescriptions and optimized the prescription structure under the AWaRe classification in the respiratory department of a county hospital, largely achieving the predefined study objectives. In addition, the patient-centered individualized education program established in this study provides a feasible practical pathway and research prospect for integrating refined antimicrobial stewardship measures into routine clinical care on a regular basis. Nevertheless, this study has several limitations. It adopted a before-and-after design across the same seasons of different years without a strict concurrent parallel control group; thus, the impacts of confounders such as seasonal variation and routine hospital quality control cannot be fully isolated. Stratified health education according to patients’ educational attainment was not implemented, and the independent effects of health education and the intelligent tool cannot be distinguished, which partly limits the generalizability of the findings. Future studies are recommended to adopt parallel controlled designs, deliver stratified and targeted interventions based on population characteristics, and further quantify the individual contribution of each measure, so as to generate higher-level evidence for antimicrobial stewardship in primary medical institutions.

## Data Availability

The original contributions presented in the study are included in the article/Supplementary material, further inquiries can be directed to the corresponding author/s.

## References

[1] James R Hardefeldt LY Ierano C Charani E Dowson L Elkins S . Antimicrobial stewardship from a one health perspective. Nat Rev Microbiol. (2026) 24:146–62. doi: 10.1038/s41579-025-01233-3, 40931120

[2] Fan Z Zhang Y Wu Y Yin J Sun Q. Public understanding and misuse of antibiotics in China: insights from a comprehensive national survey. Lancet Reg Health West Pac. (2025) 62:101682. doi: 10.1016/j.lanwpc.2025.101682, 41018943 PMC12464692

[3] Antimicrobial Resistance Collaborators. Global burden of bacterial antimicrobial resistance in 2019: a systematic analysis. Lancet. (2022) 399:629–55. doi: 10.1016/S0140-6736(21)02724-0, 35065702 PMC8841637

[4] Natadidjaja RI Lekok W Marja TS Adlani H Ariyani A Mulyani S . The appropriateness of initial intravenous empirical antibiotic consumption guided by digital antimicrobial stewardship tool: a correlation with the usage of antibiotics from the access and watch groups in ten Indonesian hospitals. Front Digit Health. (2026) 8:1862829. doi: 10.3389/fdgth.2026.1862829, 42591346 PMC13461712

[5] Natadidjaja RI Ariyani A Adlani H Adianto R Pertiwi II Legoh GN . A survey on define daily dose of watch- and access-category antibiotics in two Indonesian hospitals following the implementation of digital antimicrobial stewardship tool. Clin Ehealth. (2024) 7:176–89. doi: 10.1016/j.ceh.2024.12.004

[6] Zhao H Wei L Li H Zhang M Cao B Bian J . Appropriateness of antibiotic prescriptions in ambulatory care in China: a nationwide descriptive database study. Lancet Infect Dis. (2021) 21:847–57. doi: 10.1016/S1473-3099(20)30596-X, 33515511

[7] Xu J Liu J Li X Zhao L Shen J Xia X. Burden of bacterial antimicrobial resistance in China: a systematic analysis from 1990 to 2021 and projections to 2050. J Adv Res. (2026) 81:809–22. doi: 10.1016/j.jare.2025.06.021, 40513656 PMC12957829

[8] Wang J Wang P Wang X Zheng Y Xiao Y. Use and prescription of antibiotics in primary health care settings in China. JAMA Intern Med. (2014) 174:1914–20. doi: 10.1001/jamainternmed.2014.5214, 25285394

[9] Wu Y Zhang Z Zhao N Yan Y Zhao L Song Q . Primary health care in China: a decade of development after the 2009 health care reform. Health Care Sci. (2022) 1:146–59. doi: 10.1002/hcs2.14, 38938556 PMC11080909

[10] Qi W Zhang W Tong J Chen Z. Development of a nomogram for predicting the risk of potentially inappropriate medication use in older adults: a retrospective cross-sectional study. J Int Med Res. (2026) 54:1443912. doi: 10.1177/03000605261443912, 42082428 PMC13157572

[11] Wei X Zhuo C Hicks JP Zhang Z Wu S Walley JD . Effects of a comprehensive antibiotic stewardship program on antibiotic prescribing for acute respiratory infections in rural facilities: a cluster randomized trial. Nat Med. (2026) 32:841–8. doi: 10.1038/s41591-026-04222-y, 41703293

[12] Probst S Saini C Bobbink P Frei A Dudley-Martin F Gafner S . The impact of a multidisciplinary patient education program on venous leg ulcer healing: a randomised controlled trial. Wound Repair Regen. (2025) 33:e70084. doi: 10.1111/wrr.70084, 40880420 PMC12396614

[13] Mash R Kroukamp R Gaziano T Levitt N. Cost-effectiveness of a diabetes group education program delivered by health promoters with a guiding style in underserved communities in Cape Town, South Africa. Patient Educ Couns. (2015) 98:622–6. doi: 10.1016/j.pec.2015.01.005, 25641665

[14] Yue XH Yang L Zhong JJ Liu HM Wang D Tao X . Optimization and impact of an evidence-based pre-audit prescription decision system in primary healthcare settings. Front Pharmacol. (2025) 16:1491810. doi: 10.3389/fphar.2025.1491810, 40297139 PMC12034548

[15] Xiao Y Yu W Xiao T Shen P. Evaluating the integrated antimicrobial stewardship system of China by the assessment tool of WHO. Health Care Sci. (2022) 1:69–85. doi: 10.1002/hcs2.16, 38938893 PMC11080710

[16] National Health Commission of the People's Republic of China. *Guiding Principles of Clinical Use of Antibiotics* (2015). Available online at: http://www.gov.cn/xinwen/2015-08/27/content_2920799.htm. (Accessed July 24, 2015).

[17] Acampora M Guasconi M Schiroli C Coschignano C Cassinari N Cipolla R . Uncovering doctors' perceived barriers and facilitators of antibiotic prescribing behaviours: a qualitative study using the theoretical domains framework. Acta Biomed. (2023) 94:e2023265. doi: 10.23750/abm.v94i6.15232, 38054668 PMC10734243

[18] Ma Y Huang K Liang C Mao X Zhang Y Zhan Z . Real-world antibiotic use in treating acute exacerbations of chronic obstructive pulmonary disease (AECOPD) in China: evidence from the ACURE study. Front Pharmacol. (2021) 12:649884. doi: 10.3389/fphar.2021.649884, 34113250 PMC8185337

[19] Yang YY Ling KX Zhang X Du KX Zhang WM Hu L . Comparative analysis of domestic classification management list for clinical use of antibiotics versus WHO AWaRe classification list of antibiotics. China Pharm. (2022) 33:2945–51. doi: 10.6039/j.issn.1001-0408.2022.24.01

[20] Xu M Wu J Wang T Qiu C Zhao Y Li H . Effectiveness of doctors' advice on non-prescription antibiotic use: a randomized controlled trial, China. Bull World Health Organ. (2026) 104:28–38. doi: 10.2471/BLT.25.293840, 41409103 PMC12706762

[21] Hunter CR Owen K. Can patient education initiatives in primary care increase patient knowledge of appropriate antibiotic use and decrease expectations for unnecessary antibiotic prescriptions? Fam Pract. (2025) 42:cmae047. doi: 10.1093/fampra/cmae047, 39295113 PMC11878379

[22] Salm F Ernsting C Kuhlmey A Kanzler M Gastmeier P Gellert P. Antibiotic use, knowledge and health literacy among the general population in Berlin, Germany and its surrounding rural areas. PLoS One. (2018) 13:e0193336. doi: 10.1371/journal.pone.0193336, 29474470 PMC5825110

[23] Northey A McGuren T Stupans I. Patients' antibiotic knowledge: a trial assessing the impact of verbal education. Int J Pharm Pract. (2015) 23:158–60. doi: 10.1111/ijpp.12136, 25040636

[24] Yan Y Yang J Lu Y Cui Z Chang Y. Long-term outcomes of a health information system-based feedback intervention study of antimicrobial prescriptions in primary care institutions: follow-up of a randomized cross-over controlled trial. Infect Drug Resist. (2025) 18:61–76. doi: 10.2147/IDR.S492367, 39803305 PMC11720990

[25] Duan L Liu C Wang D Lin R Qian P Zhang X . The vicious cycle of the public's irrational use of antibiotics for upper respiratory tract infections: a mixed methods systematic review. Front Public Health. (2022) 10:985188. doi: 10.3389/fpubh.2022.985188, 36339167 PMC9632431

[26] Bolton WJ Wilson R Gilchrist M Georgiou P Holmes A Rawson TM. The impact of artificial intelligence-driven decision support on uncertain antimicrobial prescribing: a randomised, multimethod study. Lancet Digit Health. (2025) 7:100912. doi: 10.1016/j.landig.2025.100912, 41372053

[27] Zhang T Shen X Chai J Liu R Wang D Yardley L . Implementing multi-component intervention to reduce antibiotic prescribing in primary care of rural China: a qualitative process evaluation of the trial. BMJ Open. (2026) 16:e108618. doi: 10.1136/bmjopen-2025-108618, 41545046 PMC12815065

