# Supplementary material for: Survey of public health education combined with intelligent prescription intervention system on antibiotic use in the county-level hospitals
Source: Front Public Health. 2026 Sep 30;14:1963236. doi: 10.3389/fpubh.2026.1963236 (PMC13661982; doi:10.3389/fpubh.2026.1963236)
Supplement: Supplementary file 1 [file Data_Sheet_1.ZIP › Revised SI.doc]

**Survey of Public Health Education Combined with Intelligent Prescription Intervention System on Antibiotic Use in the County-Level Hospitals**

Xiaoyan XIE^1^, Lei YANG^1*^, Xiaohui YUE^1^, Ailing DING^2^, Jingjing ZHONG^1*^

^1^Department of Pharmacy, Jianyang People's Hospital, Jianyang 641400, China.

^2^School of Software Engineering, Chengdu University of Information Technology, Chengdu 610225, China.

*Correspondence: Lei YANG; E-mail addresses: yanglei7099@163.com; Jingjing ZHONG; E-mail addresses: jingjing7099@outlook.com.

**Supplementary Fig. 1 A series of educational leaflets on COPD.** (A) The impact of smoke exposure; (B) Management of concomitant diabetes and COPD; (C) Sleep and COPD; (D) Summer Management strategies for COPD; (E) Dietary advice; (F) Rehabilitation exercises for elderly COPD patients.

**Supplementary Fig. 2 A series of patient education leaflets on rational antimicrobial use.** (A) Hospital acquired pneumonia; (B) Urinary tract infections; (C) Acute cholecystitis; (D) Skin and soft tissue infections.

**Supplementary Fig. 3 A series of educational articles was disseminated via the institutional WeChat.** (A) Evidence‑based preventive measures during the influenza peak season; (B) Differential diagnosis of cold‑induced nasal symptoms; (C) Clinical differentiation between influenza and the common cold, with emphasis on appropriate management; (D) Identification of viral versus bacterial etiologies in upper respiratory infections; (E) The prudent use of antimicrobial agents during influenza outbreaks; and (F) The importance of continued monitoring for secondary bacterial infections after fever resolution.

**Supplementary Fig. 4 A series of educational videos on intravenous infusion safety.** (A) Understanding COPD and dispelling related myths; (B) Essential precautions during IV infusiont; (C) The potential consequences of improper infusion practices; (D) The significance of appropriate infusion rate control and its impact on therapeutic outcomes.

**Supplementary Table 1. Monthly proportions of PPIDS alert level 5-8 before and after intervention**

|  | Before intervention  (‰, prescriptions/total prescriptions) | | | After intervention (‰, prescriptions/total prescriptions) | | |
| --- | --- | --- | --- | --- | --- | --- |
|  | 2024-10 | 2024-11 | 2024-12 | 2025-10 | 2025-11 | 2025-12 |
| Level 5 | 51.04 | 43.30 | 39.11 | 13.71 | 27.53 | 18.04 |
| Level 6 | 0.79 | 1.50 | 0.66 | 2.14 | 3.59 | 2.54 |
| Level 7 | 4.88 | 9.59 | 7.61 | 10.71 | 11.22 | 10.06 |
| Level 8 | 3.15 | 11.69 | 8.79 | 20.99 | 20.05 | 16.54 |

**Supplementary Table 2 Monthly proportions of different PPIDS alert types triggered before and after intervention**

|  | Before intervention  (‰, prescriptions/total prescriptions) | | | After intervention (‰, prescriptions/total prescriptions) | | |
| --- | --- | --- | --- | --- | --- | --- |
|  | 2024-10 | 2024-11 | 2024-12 | 2025-10 | 2025-11 | 2025-12 |
| Dosage | 19.85 | 15.28 | 17.85 | 11.71 | 10.62 | 10.52 |
| Management regulations | 2.52 | 3.90 | 3.15 | 11.57 | 13.92 | 10.75 |
| Repeat | 4.25 | 3.60 | 4.73 | 1.14 | 0.90 | 0.93 |
| Route of administration | 10.71 | 12.88 | 10.24 | 2.57 | 2.54 | 1.97 |
| Drug interactions | 5.04 | 6.14 | 3.68 | 1.57 | 1.80 | 1.16 |
| Contraindications | 13.55 | 11.09 | 12.08 | 3.57 | 6.58 | 7.40 |
| Indications | 2.05 | 2.55 | 3.15 | 1.00 | 0.60 | 0.58 |

**Supplementary Table 3 The monthly AUD before and after the intervention**

|  | Before intervention（DDDs） | | | After intervention（DDDs） | | |
| --- | --- | --- | --- | --- | --- | --- |
|  | 2024-10 | 2024-11 | 2024-12 | 2025-10 | 2025-11 | 2025-12 |
| AUD of Antibiotics（DDDs） | 136.98 | 129.06 | 118.88 | 89.86 | 89.59 | 91.84 |
| AUD of Watch（DDDs） | 59.13 | 55.95 | 49.44 | 10.61 | 18.67 | 14.18 |
| AUD of Reserve group（DDDs） | 41.11 | 42.15 | 36.81 | 4.01 | 14.73 | 9.99 |
